# Planar and Homeotropic Liquid Crystal Alignment on 3D-Nanoprinted Layers and Microstructures

**DOI:** 10.1021/acsami.6c00485

**Published:** 2026-03-13

**Authors:** Monika Halendy, Sławomir Ertman

**Affiliations:** 49566 Warsaw University of Technology, Faculty of Physics, Koszykowa 75, Warsaw 00-662, Poland

**Keywords:** 3D nanoprinting, two-photon polymerization, direct laser writing, liquid crystals, liquid crystal alignment, three-dimensional microstructures

## Abstract

Precise control of liquid crystal (LC) alignment is essential for most LC-based applications and is typically achieved using alignment layers that induce molecular orientation through surface topography or chemical interactions. Although two-photon polymerization (2PP)-based direct laser writing (DLW) has previously been explored for fabricating such layers, existing studies have largely focused on flat surfaces designed for patterned planar alignment, where LC orientation is governed by surface topography. Consequently, one of the key advantages of this technique, which is the fabrication of arbitrary three-dimensional geometries with nanoscale precision, has remained largely unexplored for LC alignment. In this work, we investigate 2PP-based DLW as a versatile fabrication platform for engineering LC alignment through the combined use of surface topography, material chemistry, and three-dimensional geometry. We first demonstrate patterned planar-homeotropic alignment on a single substrate by integrating topographical and chemical alignment mechanisms. The alignment concept is then extended beyond flat surfaces to three-dimensional microstructures, including inclined prism-like geometries, capillaries, and fully 3D-printed cells in which both alignment layers and spacers are fabricated in a single process. This approach enables controlled twisted nematic configurations without the need for postassembly substrate alignment. Furthermore, we show that arbitrary 3D-nanoprinted microstructures can be chemically functionalized with conventional alignment agents, providing additional means of tailoring LC orientation. By combining the tunable optical properties of liquid crystals with the ability of 3D nanoprinting to fabricate arbitrary three-dimensional architectures, this approach may enable the future development of microstructures that serve specific functions while simultaneously acting as alignment components.

## Graphical abstract

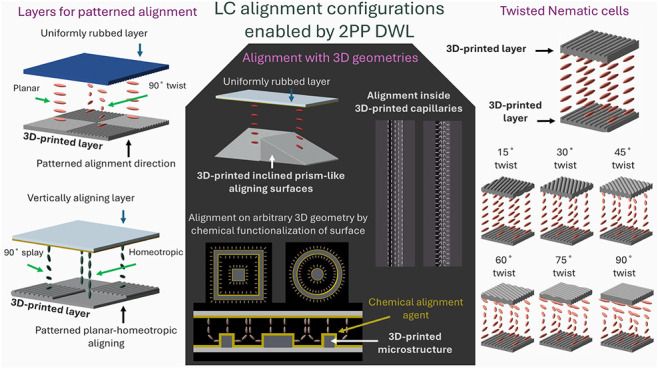

## Introduction

Controlled liquid crystal (LC) alignment is a prerequisite for the operation of most LC-based devices. Molecular ordering is necessary to exploit the anisotropic properties of LCs, which are the foundation of their technological applications.^1–3^ Such ordering is typically achieved by engineering interfacial interactions between the LC molecules and the surface of the aligning substrate, promoting their orientation in a desired direction.^4–6^ The orientation of nematic rod-like molecules can be controlled by surface topography or by surface chemistry. In the former case, the LC orientation is dictated by surface structure or shape. For instance, a polymer layer deposited on a substrate can be mechanically rubbed to produce parallel grooves in one specific direction. The molecules then orient along the rubbed grooves to minimize the elastic energy, what produces a uniform planar orientation (parallel to the surface).^7–10^ In the latter case, LC orients due to specific chemical interactions with the surface, typically achieved by depositing a tailored alignment agent onto the surface.^11–14^ It is a popular technique for obtaining uniform homeotropic orientation (normal to the surface). In most practical cases, both mechanisms coexist with different relative contributions, and the resulting LC orientation reflects their interplay, where one mechanism can be dominating.

Numerous well-established techniques are available for producing surfaces that impose a desired alignment of LC molecules, although most of these methods result in uniform alignment across the entire layer. One of the primary goals of research in this field is to achieve increasingly sophisticated alignment schemes, which can expand the potential applications of LCs. In contrast to conventional uniform alignment, patternable alignment enables the local control of LC orientation, allowing different molecular orientations in different regions of a cell.^15–19^ To achieve patterned alignment, one of the most common techniques is photoalignment, in which specific regions of a photosensitive alignment layer are illuminated with polarized light, with the polarization direction varying across different regions.^20–23^ The molecules in the alignment layer reorient according to the local polarization direction and, through their interaction with LC molecules, impose a corresponding alignment on the LC. Patterned alignment can also be realized with topographical methods, such as e-beam lithography, UV photolithography followed by nanoimprinting, and atomic force microscopy.^24–26^

Recent studies have shown that nanoscale 3D-printing technique based on two-photon polymerization (2PP) can provide a powerful method for patterned liquid crystal alignment.^27–31^ It is a direct laser writing (DLW) technique that involves scanning a focused laser beam through the volume of a photosensitive resin, inducing polymerization in the illuminated regions.^32–34^ The scanning lines produce grooves in the polymerized surface, along which the LC molecules will align. By printing multiple adjacent layers with differently oriented grooves, patterns with spatially varying LC orientations can be produced on a single substrate. The laser can also be scanned along arbitrary paths to create complex patterns, such as concentric circles or squares.^35,36^ Moreover, a PDMS-like printable material is available for the 2PP DLW technique, enabling homeotropic alignment through chemical interactions.^37–40^

In addition to the pattern freedom, such printed layers allow precise control of the anchoring energy. Previous studies have shown that varying the spacing of the scan lines leads to a linear dependence of the anchoring energy.^28,35^ Electro-optic switching behavior has also been studied, demonstrating that this alignment technique is suitable for fabricating devices such as spatial light modulators, polarization modulators, and beam-steering components.^27,29^

However, the full potential of 2PP-based 3D printing for LC alignment applications remains largely unexplored. To date, most studies have focused on flat anchoring layers, whereas 3D printing enables the fabrication of truly three-dimensional structures with nanoscale precision. Owing to these advantages, 2PP DLW has been established as a powerful tool for fabrication of microstructures, such as optical microcomponents, microsensors, microelectronics, microrobots and cell microscaffolds for biomedical applications.^41–44^ Therefore, combining the tunable optical properties of liquid crystals with the capability of arbitrary three-dimensional geometry fabrication of 3D nanoprinting enables fabrication of tunable microstructures that serve specific functions while simultaneously acting as alignment components. One of the first examples has recently been demonstrated by Vellaichamy et al.,^45^ where 2PP DLW was used to print microscale laser cavities for infiltration with a dye-doped chiral LC to serve as the active medium. Various LC-filled cavity geometries were demonstrated, including parallel and cascaded cavities, annular cavities, and single- and dual-cavity ring microlasers. Moreover, the range of potential applications is further expanded by the fact that commercially available 2PP DLW systems allow printing on unconventional substrates, such as tips of optical fibers and photonic chips. This capability may enable the future integration of 3D-nanoprinted LC devices directly into optical systems.

The study begins with an investigation of patterned flat layers, beginning with those exhibiting exclusively planar orientation, in which the direction of LC alignment varies spatially, and then progressing to layers containing patterned regions of planar and homeotropic alignment. The alignment concept is then extended beyond planar geometries to three-dimensional microstructures. Liquid crystal cells, prism-like, and cylindrical microstructures were fabricated as examples of how LC orientation can be guided in 3D geometries. The study further introduces an aligning agent coating on the microstructures, adding an extra level of control over liquid crystal alignment. The presented proof-of-concepts demonstrate the versatility of 2PP DLW as a fabrication platform for LC alignment.

## Results and Discussion

### 3D-Nanoprinted Layers for Patterned Planar Alignment

Patterned planar alignment on flat 3D-nanoprinted surfaces represents the most established and well-understood application of 2PP-based DLW for liquid crystal alignment. As such, it provides a natural starting point for evaluating the capabilities of this fabrication approach. In this section, we examine planar alignment induced by laser-written surface topography on flat 3D-printed layers, focusing on spatially varying alignment directions defined by the printing parameters.

The aligning layers were fabricated using a commercial 2PP DLW system, *Photonics Professional GT2* (*Nanoscribe GmbH & Co.*), which utilizes a femtosecond laser to scan a photosensitive resin and initiate polymerization along the scanned path. This process results in grooves formed in polymerized layers corresponding to the laser trajectory, which can then serve as a topographical aligning mechanism for LC molecules. The size of the grooves depends on a parameter commonly referred to as the hatching distance, which can be adjusted in the printer’s software (*DeScribe*, developed by *Nanoscribe*—Figure 1A). It has been demonstrated that a hatching distance of 0.3 μm or greater is sufficient to produce planar alignment along the scanning direction, with the anchoring energy increasing linearly as the hatching distance increases.^35^ Furthermore, the hatching direction can be varied across different regions of the layer using the printer’s software, offering a simple method for producing patterned planar alignment. The layer thickness is defined by the CAD model, while another printing parameter, the slicing distance (Figure 1B), determines the accuracy of the printed layer thickness.

**Figure 1:**
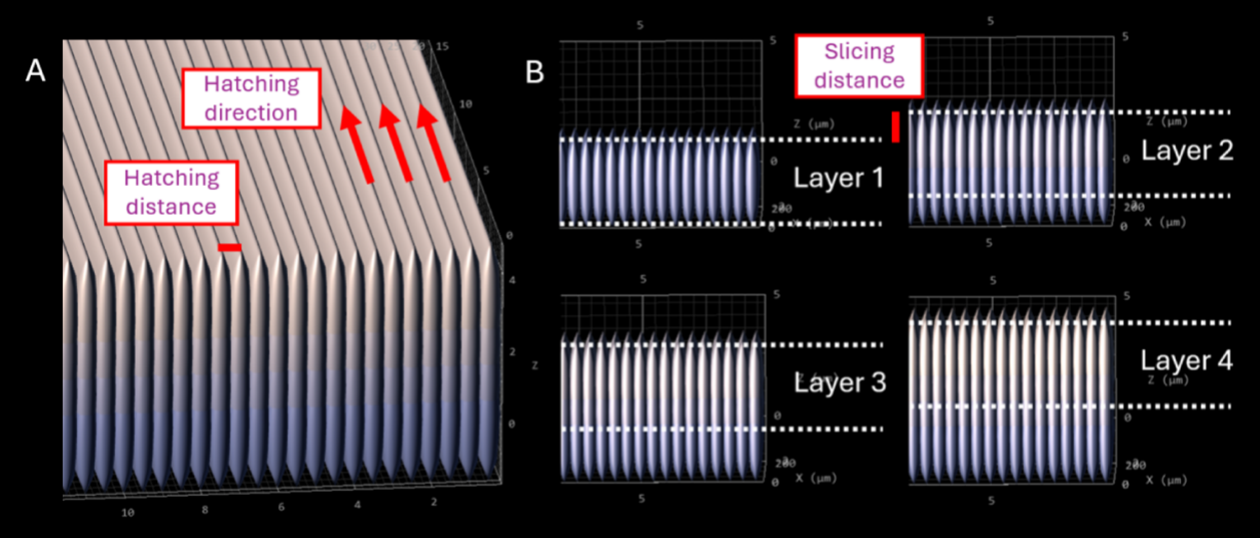
Exemplary 3D-nanoprinted layer in the printer’s software. (A) Hatching distance and hatching direction, which determine the groove’s spacing and orientation, respectively. (B) Slicing distance, which controls the vertical accuracy of the printed layer thickness.

Various strategies have been explored in the literature to achieve controlled planar alignment in liquid crystal layers. Hatching lines, which are a natural consequence of laser scanning, can serve as a topographical alignment mechanism, whereas additional topographical features are sometimes incorporated at the CAD design stage. These features can take the form of microchannels, whose depth, width, and width-to-spacing ratio can be precisely defined, thereby allowing more precise tuning of the anchoring energy compared to relying solely on hatching lines. In this study, we rely solely on hatching lines with a hatching distance of 0.5 μm. All layers for planar anchoring were fabricated using IP-S resin (*Nanoscribe GmbH & Co.*) as described in the Materials and Methods. For all experiments, the nematic liquid crystal 5CB was used.

Exemplary layers for patterned planar alignment are presented in Figure 2. A simple checkerboard-like alignment layer was fabricated and assembled into a cell of 30 μm-thick with a top substrate coated with a standard uniformly rubbed alignment layer. In this configuration, regions of the cell above the patterned layer exhibit different director orientations: some regions maintain planar alignment consistent with the hatching direction, while neighboring regions rotate by 90°, forming a local twist. As a result, when observed under polarized optical microscopy (POM), regions with directors aligned parallel to the polarizers appear dark, whereas regions exhibiting a twisted director configuration rotate the light’s polarization and appear bright, as shown in Figure 2A­(ii).

**Figure 2:**
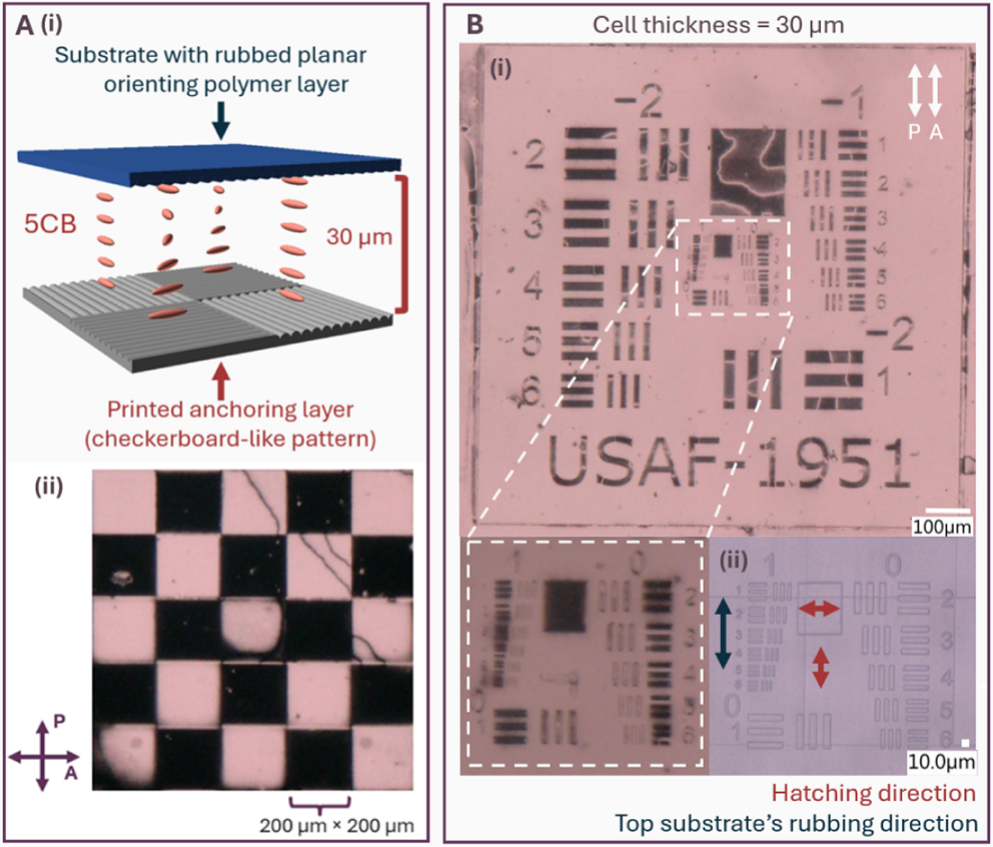
Patterned planar alignment layers fabricated using 3D nanoprinting. (A) Checkerboard-like pattern: (i) schematic of expected LC alignment in a cell with a uniformly rubbed top substrate, (ii) corresponding POM image. (B) USAF-1951 pattern: (i) POM image with the inset highlighting discrepancies between horizontal and vertical rectangular bars of the test target, (ii) digital microscope image of a printed patterned layer before infiltration with 5CB.

Using the same approach, a more complex pattern—a USAF-1951 test target—was produced (Figure 2B), in which the finest features measured 20 μm × 5 μm (Figure 2B­(i)). The rectangular bars and numbers in the target exhibited a hatching direction orthogonal to that of the background, analogous to the checkerboard pattern shown in Figure 2A. In Figure 2B­(ii), arrows indicate the hatching direction in specific regions relative to the rubbing direction of the top substrate. The bars and numbers of the target were then in a configuration to induce a 90° twist of the molecules, while the background region promoted a uniform planar alignment. Consequently, after filling the cell with LC and observing it under parallel polarizers (Figure 2B­(i)), light was transmitted in the background regions, while the regions that rotated the polarization by 90° appeared dark. Notably, while the planar alignment regions were uniform, the twist regions exhibited disclination lines, indicating the formation of domains with opposite twist handedness. It can also be observed that some domains appear darker than others. It may arise from slight deviations of the twist angle from the ideal 90°, for example 89° versus 91°.

Another conclusion may be drawn from the inset of Figure 2B­(i), where the horizontal bars, which are wider along the hatching direction of the printed layer, appear dark, whereas the vertical bars, which are narrower along the rubbing direction, remain mostly bright. This indicates that the influence of the top substrate dominates over the anchoring of the vertical bottom bars. This may be because the horizontal bars offer extended and continuous anchoring conditions along the hatching direction, while vertical bars introduce frequent interruptions of the anchoring conditions and a reduced effective anchoring area along hatching direction. In conclusion, these results demonstrate that the efficiency of LC anchoring depends on the feature geometry relative to the hatching direction. Meanwhile, Zhang et al.^28^ demonstrated that features as small as 2 μm × 2 μm can be distinguished from neighboring regions in a 15 μm-thick LC cell. In this study, experiments were conducted with a 30 μm-thick cell, which may partly explain why the smallest resolvable features were larger. Furthermore, in Zhang et al.’s study, additional CAD-designed topographical patterns were incorporated, and preliminary tests were conducted to select 3D-printing parameters that maximize anchoring energy. These observations indicate that the minimum achievable feature size is governed not only by the design of the patterned layer, but also by the specific 3D-printing parameters employed.

### 3D-Nanoprinted Twisted Nematic (TN) Cells with Varying Twist Angle

In previous studies, 3D-nanoprinted patterning was most commonly realized on a single substrate, which was subsequently assembled into a cell with a second substrate coated with an unpatterned aligning layer prepared with a conventional method. Guo et al.^36^ fabricated 3D-nanoprinted patterned layers on both substrates and, after a careful alignment process, assembled into a cell with different patterns on the top and bottom substrates. Lee et al.^30^ printed several uniform alignment layers stacked on top of one another, which were then assembled with ITO-coated glass to construct a multilayer light modulator. Here, we demonstrate a fully printed liquid crystal cell in which the alignment layers and the spacers separating them are fabricated on a single substrate (Figure 3). The bottom and top layers have different hatching directions, enabling the formation of Twisted Nematic (TN) cells with varying twist angle. The active area of these cells is 220 μm × 220 μm, although larger cells can be printed if extra supports are provided to ensure uniform cell thickness.

Figure 3A shows SEM images of printed layers with varying hatching direction. The angle was varied between 0° and 90° in 15° increments. The hatching lines are clearly visible, the spacing between them (the hatching distance) was set to 0.5 μm. For the realization of TN cells, the bottom layer had a hatching angle of 0°, while the top layer’s hatching angle varied for each cell, following the scheme shown in Figure 3B. A digital microscope image of the printed cells is shown in Figure 3C. All seven cells were fabricated on a single glass substrate in a single printing process using one script as described in Materials and Methods. The twisted alignment in the TN cells was confirmed using POM images, as shown in Figure 3D. Under crossed polarizers (Figure 3D­(i)), a gradual increase in brightness is observed corresponding to the increasing twist angle of the cell. A discernible difference in brightness is observed between two cells with a 15° difference in top-layer hatching angles. When the polarizers are rotated to 45° (Figure 3D­(ii)), the cell with a top-layer hatching angle of 45° exhibits maximum brightness, consistent with the expected LC director orientation relative to the polarizers. With parallel polarizers (Figure 3D­(iii)), the brightness pattern is inverted relative to that observed in Figure 3D­(i). Overall, these observations indicate precise control over the twist angle in the printed TN cells.

Apart from the differences in brightness, differences in uniformity can also be observed. Similar to the situation in Figure 2B (patterned USAF-1951 planar-90° twist layer), the cell with 0° twist is the most uniform, while the uniformity decreases as the twist angle increases. For the 90°-twist cell, the LC layer is strongly separated into domains. Apart from that, the color across a cell is also not fully uniform, indicating slight thickness variations, most likely due to slight deflections of the top layer. Both the bottom and top layers had a relatively robust thickness of 10 μm; for thinner top layers, deflections would likely be more pronounced. To ensure better thickness control, additional supports could be added. It is also worth noting that the rounded feature in the 90° twist cell is an air bubble introduced during the infiltration procedure.

**Figure 3:**
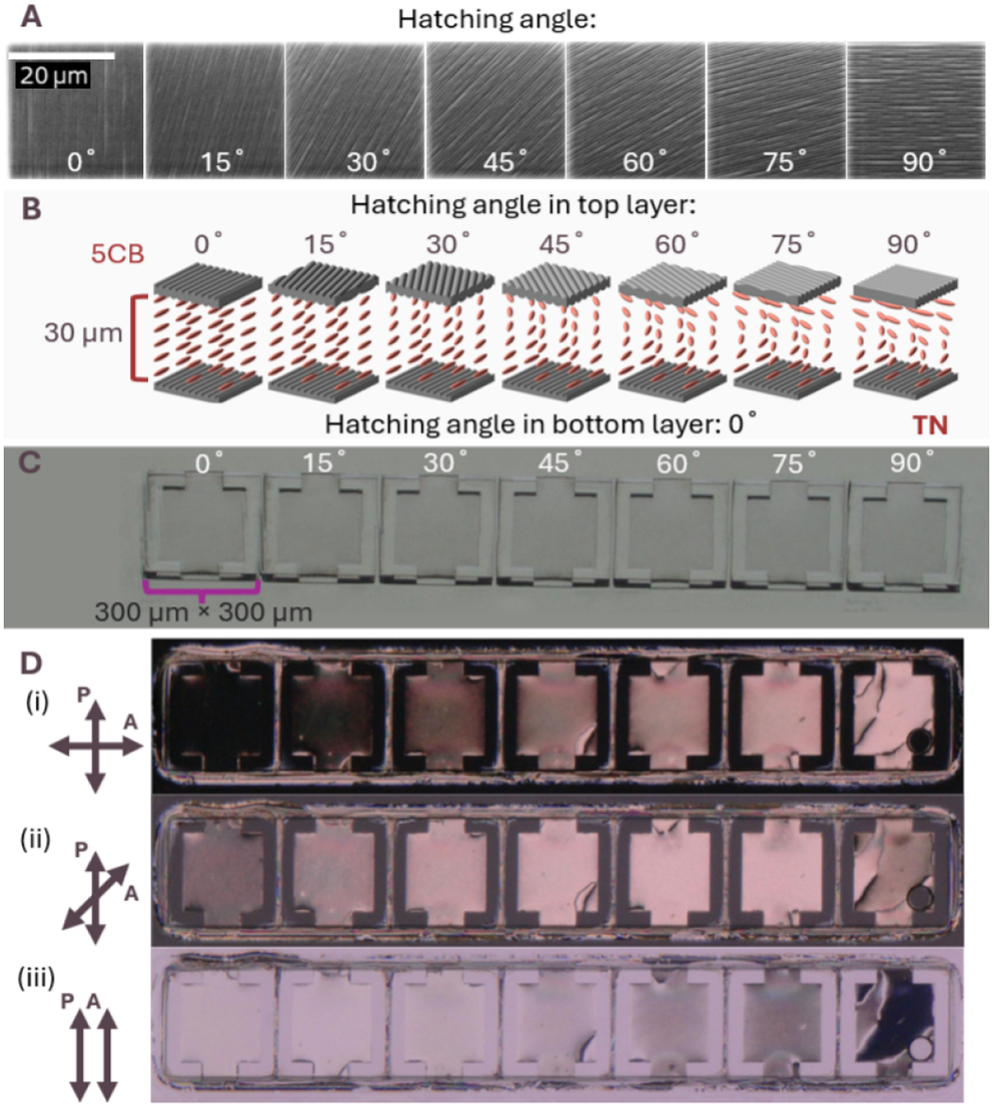
3D-nanoprinted Twisted Nematic (TN) cells. (A) SEM images of 3D-nanoprinted layers with varying hatching angles. (B) Schematic of bottom and top layers for TN cells. (C) Digital microscope image of the printed TN cells. (D) POM images of the TN cells: (i) crossed polarizers, (ii) polarizers at 45°, (iii) parallel polarizers.

This type of cells could be particularly useful for in-plane switching, in which the electric field is generated laterally between electrodes patterned on the same substrate and is therefore oriented parallel to the substrates, inducing a rotation of the LC director within the plane of the cell. An alternative switching configuration involves placing an additional electrode-bearing substrate on top of the cell. In this case, the advantage of the 3D-nanoprinted cells, compared to the approach with alignment layers printed on two separate substrates, is that no careful alignment procedure is required. Another advantage over previously reported approaches is that, whereas conventional methods for cell assembly typically involve thin Mylar foils or microbeads to control the substrate spacing, the spacers in the presented approach are 3D-nanoprinted, allowing their thickness to be chosen arbitrarily.

### 3D-Nanoprinted Layers for Patterned Planar and Homeotropic Alignment

Homeotropic alignment can be achieved through both topographical and chemical mechanisms. Topography-induced alignment can be realized with a surface exhibiting height variations in two dimensions, in such a way that it would be energetically advantageous for the molecules to line up normal to the surface rather than parallel, as the latter would require bending or other elastic deformations.^7^ One demonstrated approach involves depositing opal crystals, where the topography of the nanoparticles guides the LC’s homeotropic orientation.^46^ It has also been realized in a patternable manner by nanoimprinting with molds previously prepared by electron-beam lithography.^47^ In this method, microscale arrays of square wells with varying sizes and depths were fabricated, and the LCs were observed to align parallel to the inner faces of the wells. 3D-nanoprinting has also been employed to achieve patterned homeotropic alignment using 3D-printed nanopillars.^36^ However, this method is effective only over a narrow range of topographical parameters. For instance, the wells need to be sufficiently narrow and deep.

3D-nanoprinted layers for homeotropic alignment can also be achieved by leveraging their chemical properties. Previous studies have shown that PDMS layers can induce vertical alignment of liquid crystals, and *Nanoscribe* offers a PDMS-like photosensitive resin (IP-PDMS) suitable for this purpose. This property of the printable material has been exploited to fabricate microscaffolds of arbitrary shapes, enabling precise control of liquid crystal orientation in three-dimensional space, and facilitating the subsequent creation of deformable structures for use as actuators.^37^ This demonstrated significant potential for three-dimensional alignment. Here, we aim to highlight a different capability—patternable planar-homeotropic alignment within a single cell, achieved by creating distinct regions from two different materials used in 2PP DLW 3D-nanoprinting.

As illustrated in Figure 4, a checkerboard-like pattern was fabricated using two materials. IP-S was employed to create grooves for planar alignment, similar to the layers presented in Figures 2 and 3, while IP-PDMS induced homeotropic molecular orientation. A glass substrate coated with an SE1211 (*Nissan Chemical Corporation*) layer was used as the top substrate, a material commonly employed to promote vertical alignment. Consequently, a splay configuration was formed between the IP-S and SE1211 layers, whereas a purely homeotropic alignment was obtained between the IP-PDMS and SE1211 layers, as schematically illustrated in Figure 4A.

The fabrication of such layers requires two separate printing processes. First, structures are printed using the IP-S resin, after which the substrate must be thoroughly cleaned with a solvent sequence of propylene glycol monomethyl ether acetate (PGMEA) followed by isopropanol (IPA). Subsequently, the IP-PDMS resin was applied to the same substrate, and, after alignment under visual inspection with a camera, new layers were printed in the regions between the previously fabricated IP-S layers. After this step, the resin was removed using isopropanol only. According to the resin supplier (*Nanoscribe*), IP-S must be developed using PGMEA and IPA, whereas IP-PDMS should be developed exclusively with IPA. Consequently, the order of printing cannot be reversed.

Figure 4C shows the hybrid planar-homeotropic alignment in the printed layers. When the crossed polarizers were oriented at 45° relative to the planar alignment direction (Figure 4C­(i)), alternating dark and bright regions were observed, corresponding to homeotropic and planar alignment, respectively. After rotating the polarizers by 45°, the regions previously appearing bright became dark, confirming planar alignment, while the dark regions remained unchanged, indicating homeotropic alignment. A slight overlap of dark regions into adjacent bright regions was observed in the upper row, likely due to residual IP-PDMS from the second printing step. This effect may be mitigated in future work by optimizing the isopropanol rinsing time. Disclination lines are visible in the IP-S-SE1211 splay regions, which indicates splay degeneracy, meaning that as molecules rotate from the planar orientation at the bottom layer to the vertical orientation at the top layer, they can perform this rotation tilting toward either of two mirror-symmetric azimuthal directions.

**Figure 4:**
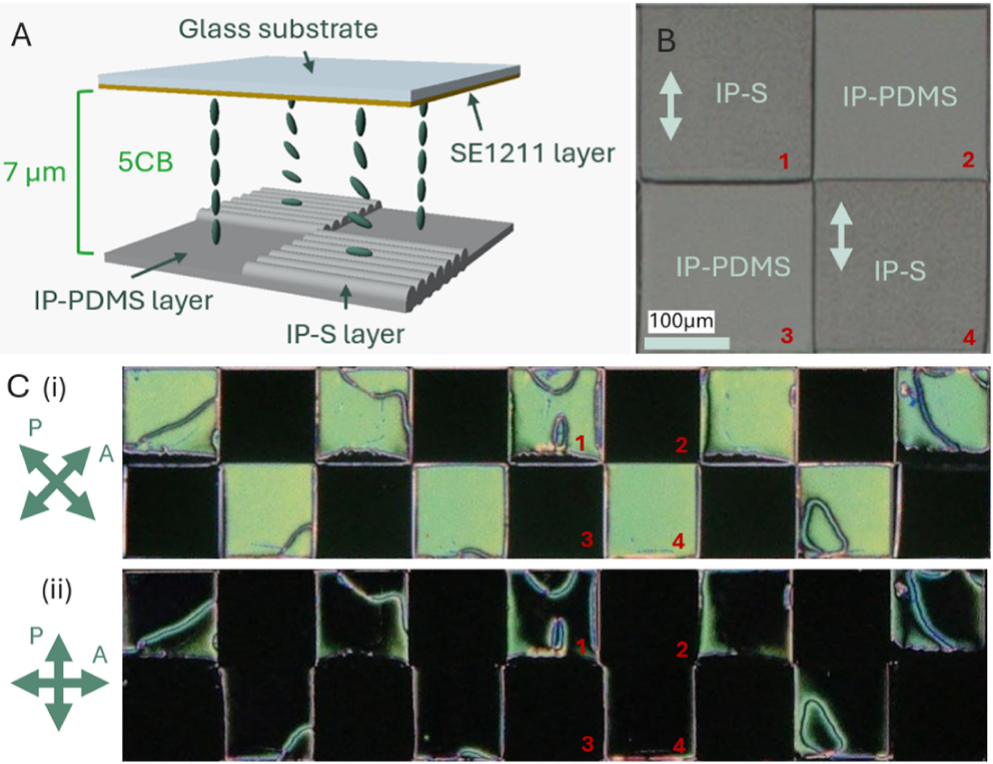
Patterned planar-homeotropic alignment realized using a checkerboard-like arrangement of two materials. (A) Schematic illustration of the alignment configuration. (B) Digital microscope image of alternating IP-S and IP-PDMS regions. (C) Polarizing optical microscopy images of the hybrid alignment under crossed polarizers: (i) polarizers oriented at 45°, (ii) polarizers oriented at 0°.

### LC Alignment on 3D-Nanoprinted Prism-Like Microstructures

The full potential of 3D-printed alignment layers lies in the ability to create three-dimensional geometries that control liquid crystal orientation. To date, this potential has primarily been exploited for the fabrication of 3D-nanoprinted tunable microlenses, which, in addition to their beam-shaping functionality, also serve as alignment surfaces.^48^ One particularly interesting demonstration is the 3D-nanoprinted walls with nanogrooves on the sides, which enable planar LC alignment along vertical or horizontal directions depending on the printing parameters, as shown by Jagodic et al.^49^ In their work, they produced these two configurations by simply adjusting the slicing and hatching distances: small slicing and large hatching to produce vertical grooves, and large slicing with small hatching to produce horizontal grooves. This architecture offers a promising approach for future microphotonics applications, as two such walls could be used to build a cell for trapping and aligning LC between them. It was demonstrated that LC could be oriented between the facing edges of two microprisms.

Here, we extend this concept by presenting LC alignment on the surfaces of prism-like microstructures, where the grooves are produced on tilted surfaces of the prisms rather than on the back walls (Figure 5A). The lateral dimensions of the patterned areas were 200 μm × 200 μm, with the height increasing up to 15 μm across the structure. Two types of layers were fabricated: one with the hatching direction perpendicular to the inclination and one with the hatching direction parallel to the inclination. A very fine slicing distance was employed, automatically adjusted by the printer’s software, to create a smoothly inclined surface, as shown in the digital microscope image in Figure 5B. When alignment along the inclination was targeted, the fine slicing distance also helped to mitigate the influence of pronounced interlayer steps, which could otherwise hinder molecular alignment along the slope. The hatching distance was maintained at 0.5 μm. The top substrate was rubbed parallel to the hatching direction to promote purely planar alignment.

POM images of the prism-like microstructures are shown in Figure 5C. The rainbow-like color variations observed in Figure 5C­(i) arise from the gradual change in the local cell thickness across the prism-like structure. However, the interference fringes are not perfectly straight but slightly rounded. This suggests that the prism geometry deviates from the ideal shape, with the tilted surface not being perfectly planar. It may originate from minor edge shrinkage or slight delamination at the structure boundaries, which was not initially apparent during digital microscope or SEM inspection but became evident under polarized optical microscopy. Upon rotating the crossed polarizers by 45°, this region becomes uniformly dark, as shown in Figure 5C­(ii). This behavior indicates planar alignment of the liquid crystal, both along and perpendicular to the tilt direction of the microstructure.

**Figure 5:**
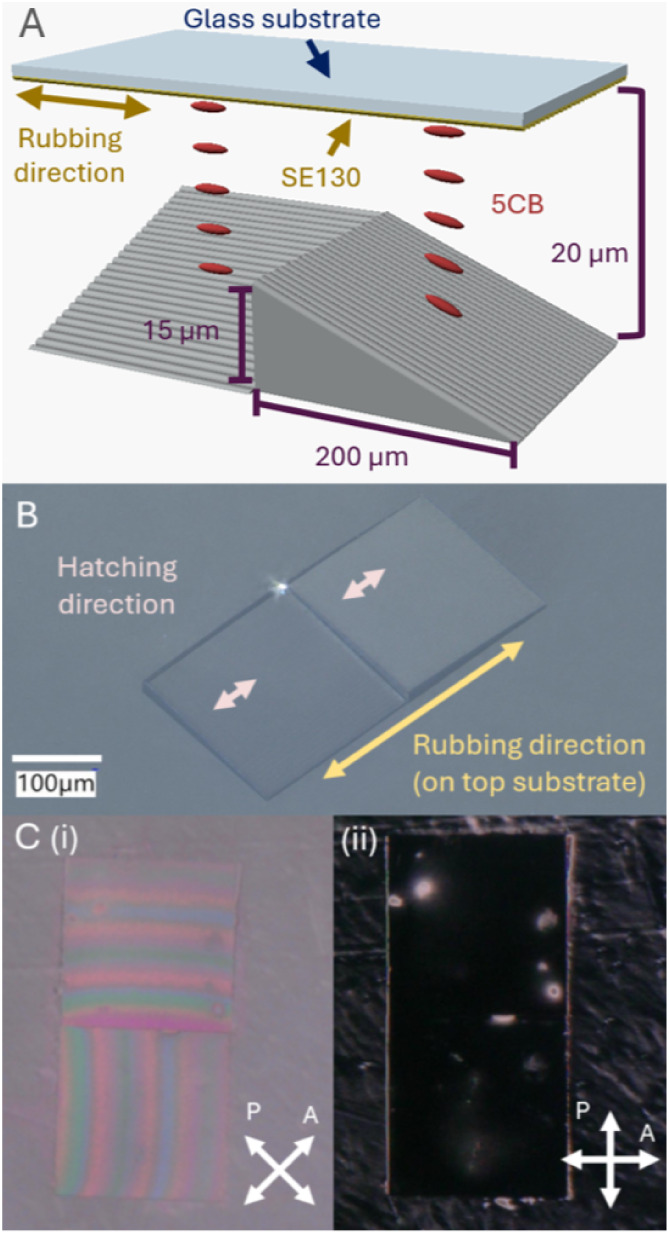
Prism-like 3D-printed microstructures for liquid crystal alignment. (A) Schematic of the microstructure geometry and molecular alignment. (B) Digital microscope image of the structure. (C) POM images: (i) crossed polarizers rotated by 45°, (ii) crossed polarizers at 0°.

### LC Alignment in 3D-Nanoprinted Capillaries

While conventional liquid crystal cells provide a well-established platform for studying surface-induced alignment, capillaries offer an alternative geometry in which confinement and curvature play a dominant role. In such cylindrical systems, molecular alignment becomes a critical factor, as the interplay between surface anchoring, elastic distortions, and flow during filling gives rise to director configurations that cannot be realized in planar cells.^50,51^

In the simplest case, planar alignment arises naturally due to capillary filling, where the flow induces orientation. Other configurations, such as splay or tilted alignment, require additional treatment: the capillaries are first filled with an aligning agent, which leaves a thin film on the inner walls after removal.^52^ Photoalignment techniques can also be employed to achieve spatially patternable alignment within capillaries.^53^

Capillaries serve not only as a valuable model system for fundamental studies of liquid crystal ordering under geometric confinement, but also find practical use as functional elements in emerging photonic and waveguiding applications.^54,55^ One example is photonic liquid crystal fibers (PLCFs), which contain capillaries in their cladding. When the capillaries are filled with LCs, they enable tunability of the propagating optical modes.^56^

In this study, we investigate 3D-nanoprinted capillaries fabricated with IP-PDMS, which induce splay alignment (Figure 6A­(iv)), as well as from IP-S to obtain planar alignment via flow-induced orientation (Figure 6B­(iv)). The splay alignment in IP-PDMS capillaries arises from the chemical anchoring properties of the material, as described in the previous section. The capillaries were printed on glass substrates and had a height of 0.5 mm, external diameter was set to 100 μm, and internal diameter was parametrized. To ensure complete infiltration, two channels were incorporated at the bottom of each capillary, allowing liquid crystals to flow from top to bottom without trapping air bubbles.

Figures 6A­(i) and (ii) show the IP-PDMS capillary with an inner diameter of 15 μm before and after filling with 5CB, respectively. In Figure 6A­(iii), the textures of IP-PDMS capillaries with three different diameters are compared under crossed polarizers at 0° and 45°. The observed dark and bright regions correspond well to the expected splay alignment illustrated in Figure 6A­(iv). Notably, discontinuities in the textures are visible, which results from vertical stitching between layers. This stitching is necessary for taller structures, as the height of the capillaries exceeds the working distance of the objective used during fabrication.

Figure 6B­(i) shows the IP-S capillary with an inner diameter of 25 μm before filling. After filling, the capillary was examined under polarized light microscopy at 0° and 45° relative to the crossed polarizers. The bright and dark regions observed in Figure 6B­(ii) and (iii) are characteristic of planar molecular orientation.

The primary advantage of 3D-nanoprinted capillaries over conventional silica capillaries lies in the ability to design arbitrary transverse geometries. It is particularly important for PLCFs, where achieving complex transverse geometries in silica fibers is difficult and costly, and typically realized only for mass production. Additionally, such waveguide segments can be printed directly onto optical fiber tips. The primary limitation of 3D-nanoprinted capillaries, however, is that only short segments on the order of a few millimeters can be easily fabricated. Nevertheless, these short segments have been demonstrated to be sufficiently long to achieve tunable performance in 3D-nanoprinted waveguides.^57,58^

**Figure 6:**
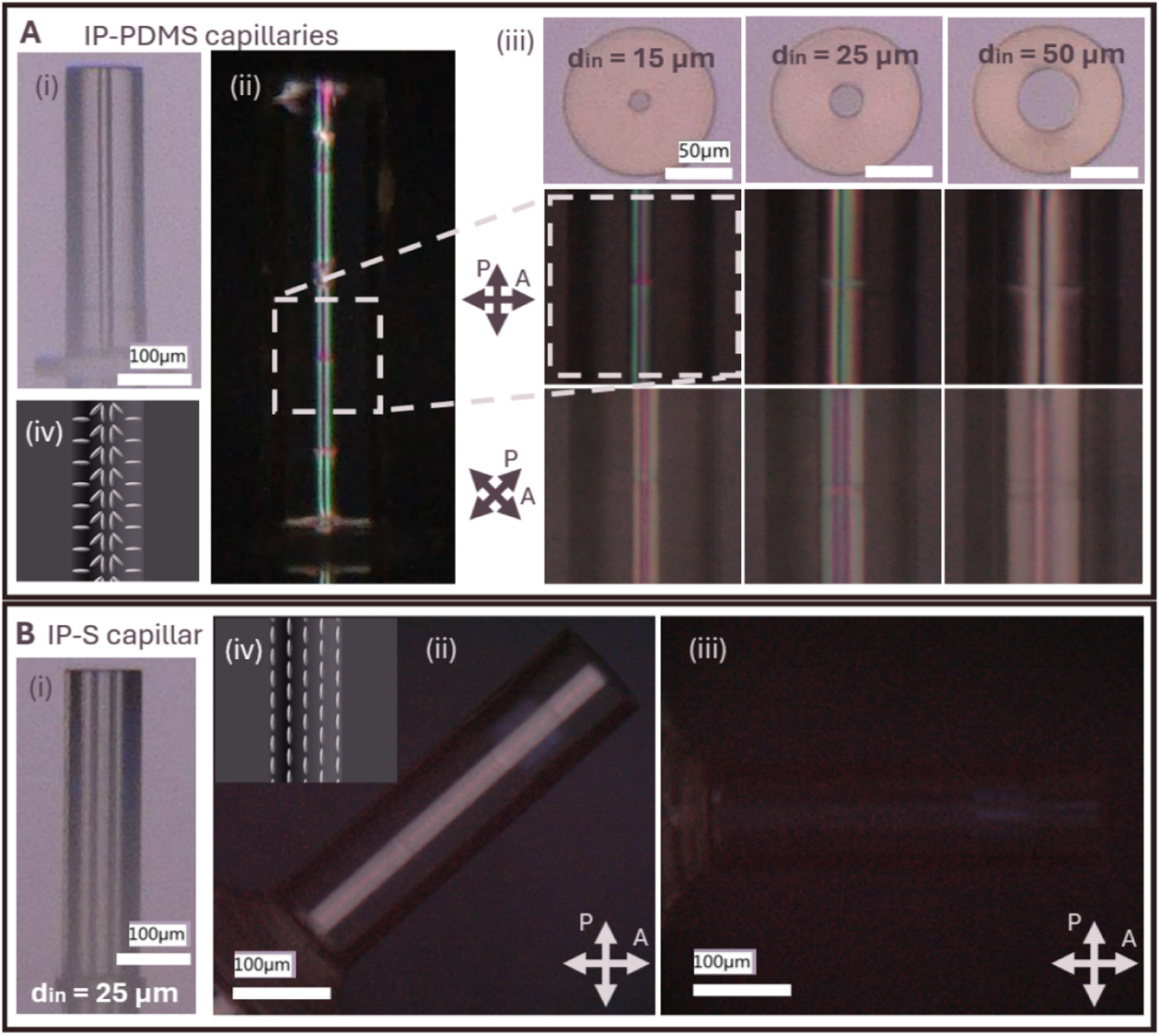
3D-nanoprinted capillaries with controlled liquid crystal alignment. (A) IP-PDMS capillaries inducing splay alignment: (i) capillary of 15 μm inner diameter before filling, (ii) after filling with 5CB, (iii) comparison of textures for three different inner diameters under crossed polarizers at 0° and 45°, (iv) schematic of splay alignment inside an IP-PDMS capillary. (B) IP-S capillary for planar alignment via flow-induced orientation: (i) capillary before filling, (ii−iii) polarized optical microscopy images under 0° and 45° relative to crossed polarizers, (iv) schematic of planar alignment inside the IP-S capillary.

### Chemical Functionalization of 3D-Nanoprinted Microstructures for LC Alignment

Liquid crystal alignment is commonly achieved using carefully designed chemical agents, which are either commercially available or can be synthesized in the laboratory.^6^ Here, we demonstrate that an agent with desired properties can be deposited onto 3D-nanoprinted microstructures as thin aligning films, thereby enabling chemical functionalization of the printed microstructures for controlled liquid crystal alignment

In this experiment, 3D microstructures with the geometry shown in Figure 7A were printed on a single glass substrate with varying heights (5 μm, 8 μm, 10 μm, and 12 μm) and different transverse dimensions *d* (80 μm, 120 μm, and 200 μm—see insets in Figure 7B). After development to remove unpolymerized resin residues, the substrate was spin-coated with SE1211, a widely used commercially available alignment agent that induces strong homeotropic anchoring. The agent is typically applied as a thin layer on glass substrates via spin coating. According to the manufacturer’s (*Nissan Chemical Corporation*) datasheet, the alignment agent should be dried at 80 °C for 5 min and then cured at 180 °C for 60 min. However, to reduce the risk of structural damage in the polymer microstructures, such as cracking or shrinkage, the curing was performed at 120 °C for 120 min. No detrimental effects on the microstructure integrity were observed under these conditions.

**Figure 7:**
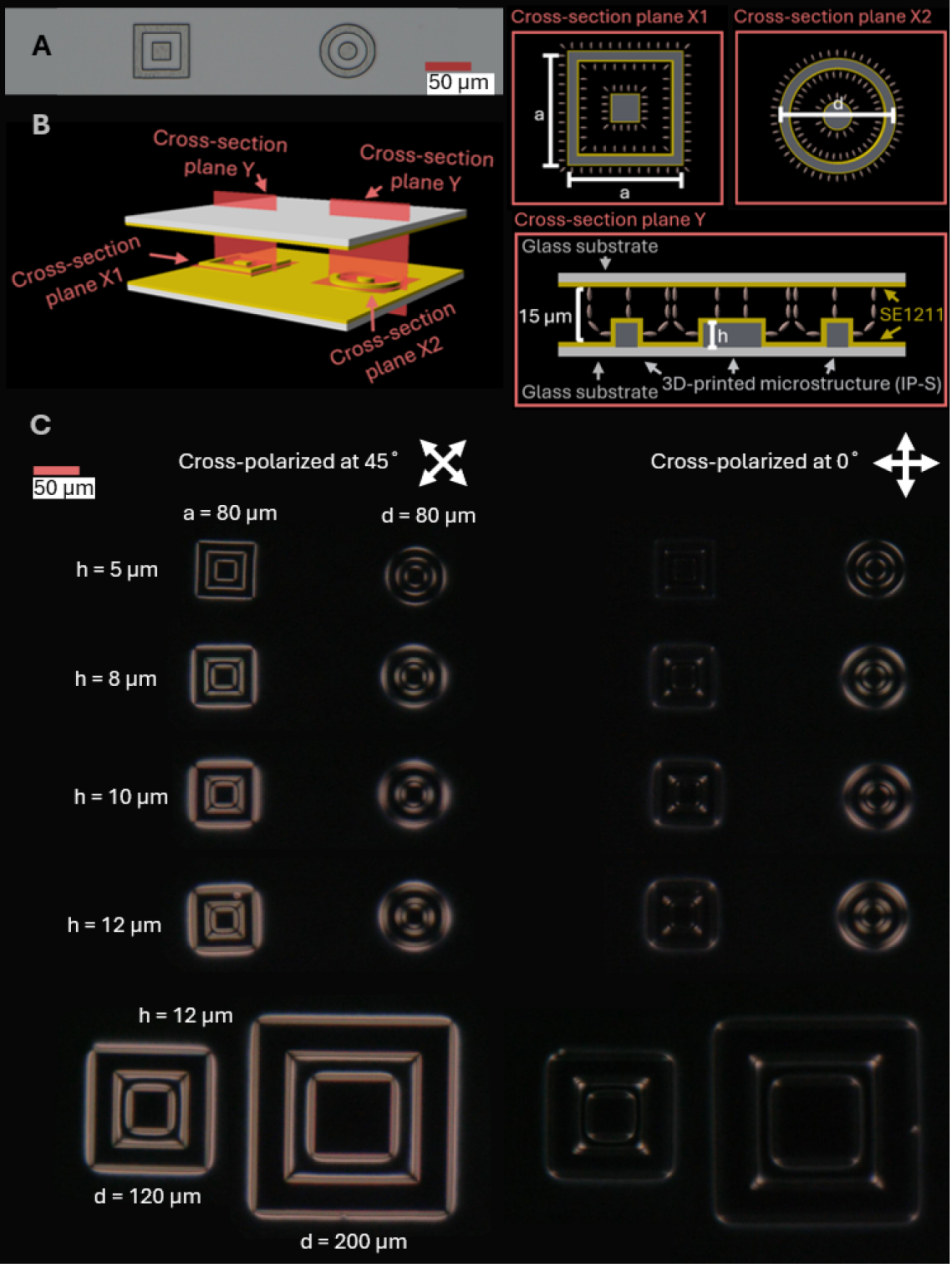
Liquid crystal alignment on 3D-nanoprinted microstructures functionalized with SE1211. (A) Digital microscope image of the printed microstructures. (B) Schematic of the printed microstructure geometry and LC alignment. (C) POM images with crossed polarizers at 45° and 0°.

The substrate with printed microstructures was then assembled with a top substrate, also coated with an SE1211 layer, forming a cell with a thickness of 15 μm. The expected liquid crystal alignment in the bulk is illustrated in Figure 7B. The cell was filled with 5CB, and POM images of the resulting alignment are shown in Figure 7C, confirming the expected orientation. An interesting effect of microstructure height can be observed: the taller the structure, the further the influence of anchoring on the side walls propagates. In contrast, varying the transverse dimensions *d* of the structures shows that, even for broader features, the propagation distance remains constant, indicating that the effect is primarily height-dependent.

These results open perspectives for the functionalization of arbitrary 3D-nanoprinted microstructures for liquid crystal alignment. In the future, this approach could be applied to both simple structures and more complex designs, such as tunable optical microresonators and waveguides for mode splitting and mode conversion.

## Applicability, Advantages, and Limitations

In this section, we would like to outline the overall applicability of 2PP DLW for liquid crystal alignment, address the key alignment properties, as well as the advantages, inherent limitations, remaining challenges, and open research gaps.

In our study, all the fabricated flat alignment layers were a few micrometers thick, which is an inherent property of the 2PP fabrication technology. More established techniques, such as spin-coating, can produce layers of ∼100 nm thickness, however achieving such thin layers with nanoprinting would be challenging. In this work, a 25× objective was used, for which the ellipsoidal voxel size typically exhibits a lateral diameter (a_*xy*_) of ∼600 nm, while its axial length (a_*z*_) is significantly larger, equal around 3 μm. *Nanoscribe* also offers a 63× objective with a lower axial length of ∼0.8 μm, however printing with this more precise objective substantially increases layer fabrication time. As a result, cells fabricated using this technique are thicker than standard ones, which may affect the required voltages for electro-optic tuning. Nevertheless, this approach provides a major advantage: it enables precise control over both the three-dimensional geometry of the structures and the local orientation of the liquid crystal molecules. Such control is difficult or impossible to achieve with conventional thin-film alignment techniques, allowing the creation of complex, spatially varying director patterns within a single cell.

Further benefits of the employed technique include high thermal stability and UV resistance, particularly compared to photoalignment methods. UV exposure actually has a beneficial effect on the 3D-nanoprinted alignment layers, as it enhances their mechanical stability by further cross-linking the polymer through 1-photon polymerization. The polymer layers and microstructures remained stable far beyond the operating temperature range of the LC used in our experiment (5CB). The polymer maintains its integrity up to temperatures of at least 100 °C. Beyond this temperature, the structures may develop cracks or lose adhesion to the glass substrate due to stress at the interface caused by different thermal expansion of the two materials. To avoid these effects when heating 3D-printed microstructures coated with SE1211, which normally requires postbaking at 180 °C, we tested lower baking temperature and 120 °C was found to be safe, although it required an extended baking time.

While the proposed alignment strategies enabled a broad range of LC configurations, not all configurations exhibited fully uniform alignment. Planar and homeotropic regions appeared highly uniform and allowed for achieving high intensity contrast (complete extinction of light under crossed polarizers). However, twisted and splayed configurations appeared to be less uniform, as disclination lines were observed. The disclination lines for splay deformations (Figure 4C) suggest that the effective pretilt angle of the 3D-nanoprinted IP-S layers was close to 0°, as introducing a small pretilt angle of ∼2° would be expected to break the degeneracy of the director configuration by imposing a preferred splay direction. The disclination lines could be avoided by slow directional cooling from isotropic phase, which would bias successive molecules to adopt the same twist or splay direction as those that have already aligned.

It is worth noting that cells incorporating complex three-dimensional microstructures are inherently characterized by pronounced local variations in surface geometry, which lead to spatial variations in tilt angle, pretilt, anchoring conditions, and other interfacial parameters. As a consequence, these quantities cannot be described by a single global value, and their direct quantitative determination at the microscale becomes nontrivial. Reliable characterization would require either highly localized probing techniques or advanced analysis of polarized optical textures acquired under different viewing angles and applied voltages.

Quantitative measurements are more readily obtained for simpler, flat nanoprinted layers. Anchoring energies of the 3D-nanoprinted flat layers for planar alignment have been reported to be in the range of $10^{-6}\frac{J}{m^{2}}$ to $10^{-5}\frac{J}{m^{2}}$ , depending on the groove geometry.^28^ These values have been measured for IP-Dip layers, although the printed layers were conceptually similar to those obtained with IP-S. For IP-S, anchoring energy was measured in an alternative printing configuration, where polymer surfaces were printed vertical to the substrate (aligning “walls”), and found to be $4\times 10^{-6}\frac{J}{m^{2}}$ .^49^ The authors concluded that the anchoring energy of the 2PP DLW printed layers can be tailored between $10^{-7}\frac{J}{m^{2}}$ and $10^{-5}\frac{J}{m^{2}}$ by changing the printing parameters.

These results demonstrate that anchoring energies can be quantified and even tailored for certain 2PP-printed polymers such as IP-S and IP-Dip, depending on printing parameters and layer geometry. However, IP-PDMS seems largely uncharacterized in this respect. It is known that traditional PDMS thin films on glass substrates can enforce vertical LC alignment, with LC molecules aligning in a near-90° tilt.^39,40^ This indicates strong homeotropic anchoring, although quantitative anchoring energy values are not reported in these works. The most popular application of IP-PDMS in 3D-nanoprinting is for fabrication of soft, deformable microstructures, and has rarely been used for LC alignment so far. Characterizing its surface anchoring behavior for LC alignment therefore constitutes an important research gap.

## Conclusions

In this work, we used 2PP-based 3D nanoprinting to combine topographical design, material chemistry, and three-dimensional geometry for LC alignment within a single fabrication platform. In the first step, patterned planar alignment was realized on flat 3D-nanoprinted layers utilizing grooves for topographical alignment. Checkerboard and USAF-1951 test patterns were realized, demonstrating precise and spatially resolved control of the LC director. Building on this capability, fully 3D-nanoprinted Twisted Nematic cells were fabricated, in which both alignment layers and spacers were printed in a single process, allowing deterministic control of the twist angle without the need for substrate alignment. Subsequently, we combined the topographical approach with chemical alignment by fabricating layers from IP-S and IP-PDMS on a single substrate and achieved hybrid planar-homeotropic alignment patterns.

The alignment concept was then extended beyond planar geometries to truly three-dimensional microstructures. Prism-like structures demonstrated controlled planar alignment on inclined surfaces, while 3D-nanoprinted capillaries enabled cylindrical confinement with chemically and flow-induced director configurations. Furthermore, we showed that arbitrary 3D-nanoprinted microstructures can be chemically functionalized with conventional alignment agents.

Overall, this work demonstrates that 3D nanoprinting can be used not only to fabricate alignment layers, but to engineer complete liquid crystal alignment architectures in three dimensions. We anticipate that this approach will enable LC-based photonic systems in which alignment, geometry, and functionality are designed simultaneously rather than sequentially.

## Materials and Methods

All layers and microstructures were fabricated using a commercial *Photonics Professional GT2* system (*Nanoscribe GmbH & Co*., Germany). All structures were first designed in CAD software and exported as STL files, which were then imported into the *Nanoscribe DeScribe* software to define printing parameters and generate JOB files interpretable by the printer. The manufacturer provides a wide range of printable resins. For this study, IP-S and IP-PDMS resins were used. A 25× objective was employed for all prints. The IP-S layers were printed with a hatching of 0.5 μm and a slicing of 1.0 μm, while IP-PDMS layers were printed with hatching and slicing distances of 0.3 μm, according to the default recipes provided by *Nanoscribe*. In all experiments, 5CB liquid crystal (*Ossila Ltd.*, UK) was used. After filling the cells, the samples were heated to the clearing temperature of 5CB (around 45 °C) and then cooled to room temperature to eliminate flow-induced alignment effects. All LC textures were analyzed using a *Keyence VHX-5000* microscope equipped with two polarizers for the visible range.

### Rinsing of 3D-Nanoprinted Layers and Microstructures

After printing, all layers and microstructures require rinsing to remove unpolymerized polymer from their vicinity. The IP-S resin should be dissolved in PGMEA, followed by rinsing in IPA, whereas IP-PDMS should be developed in IPA only, according to the datasheets. The rinsing time should be adjusted for each type of structure, with tighter regions, such as narrow gaps, requiring longer times to ensure complete infiltration of the developer.

IP-S layers and “open” microstructures (e.g., prism-like) were developed in PGMEA for 15 min, followed by rinsing in IPA for 5 min. IP-PDMS layers were developed in IPA for 15 min. For the IP-S LC cells, the rinsing time had to be extended to 45 min in PGMEA and 15 min in IPA to remove the unpolymerized resin from the narrow gaps between the bottom and top alignment layers. Similarly, for the IP-PDMS capillaries, development times were extended to 45 min in IPA and for the IP-S capillary to 45 min in PGMEA and 15 min in IPA to ensure complete removal of unpolymerized material from the interiors.

### Writing Scripts for Patterned Layers

The *DeScribe* software allows combining multiple structures with different printing parameters into a single printing process using a script. In the *Nanoscribe* system, fabrication is controlled through two GWL files: a “data” file and a “job” file. A “data” GWL file contains the machine-level writing instructions, including hatching, slicing, and laser movement paths derived from the processed geometry. A “job” GWL file functions as a higher-level container that can reference one or multiple “data” GWL files and defines global printing parameters such as writing sequence, stage movements, and exposure settings.

To combine multiple STL-defined structures, each geometry was processed separately to generate individual “data” GWL files. These files were then referenced within a single “job” file, enabling fabrication of all structures in one continuous writing process, with spatial arrangement controlled through object positioning in *DeScribe*.

To realize patterning of the hatching direction, a single STL file defining the layer geometry was imported into *DeScribe*. Multiple GWL files were then generated from the same STL file, differing only in the hatching direction assigned to each GWL. The corresponding “data” GWL files were subsequently combined within a single “job” script, enabling fabrication of regions with varying hatching directions in a single printing step. The script controlled the relative positioning of each region to ensure accurate alignment across the entire patterned structure.

### Writing Scripts for TN Cells

To print a series of seven TN cells side by side, each with a different top-layer hatching angle, in a single printing process, a custom script was written. The script was based on two STL files: one defining the geometry of the bottom layer and spacers, and one defining the top layer only (Figure 8A­(i−ii)). From the first STL, a single “data” GWL file was prepared with a hatching angle of 0° for the bottom layer. From the second STL, seven “data” GWL files were generated, each with a different top-layer hatching angle. In the “job” script, the bottom-layer’s GWL was included seven times, each with adequately shifted position along the *x*-axis. The top-layers’ GWLs were then included, with positions above the corresponding bottom layers at the appropriate Z spacing, determined by the spacer thickness. A visualization of a few exemplary script steps is shown in Figure 8A­(iii−vi).

### Preparation of LC Cells with 3D-Nanoprinted Bottom Layer and Top Glass with Commercial Alignment

The substrate with a 3D-nanoprinted layer was bonded to a substrate coated with a commercial alignment layer (provided by *Nissan Chemical Corporation* (Japan): rubbed SE130 for planar alignment and SE1211 for homeotropic alignment) with a UV-curable adhesive, which was heated and pressed to ensure uniform spreading. Cell thickness was defined by 3D-nanoprinted spacers (Figure 8B), allowing for arbitrary adjustment of the cell gap. After gluing, the cell thickness was verified using SEM images.

**Figure 8:**
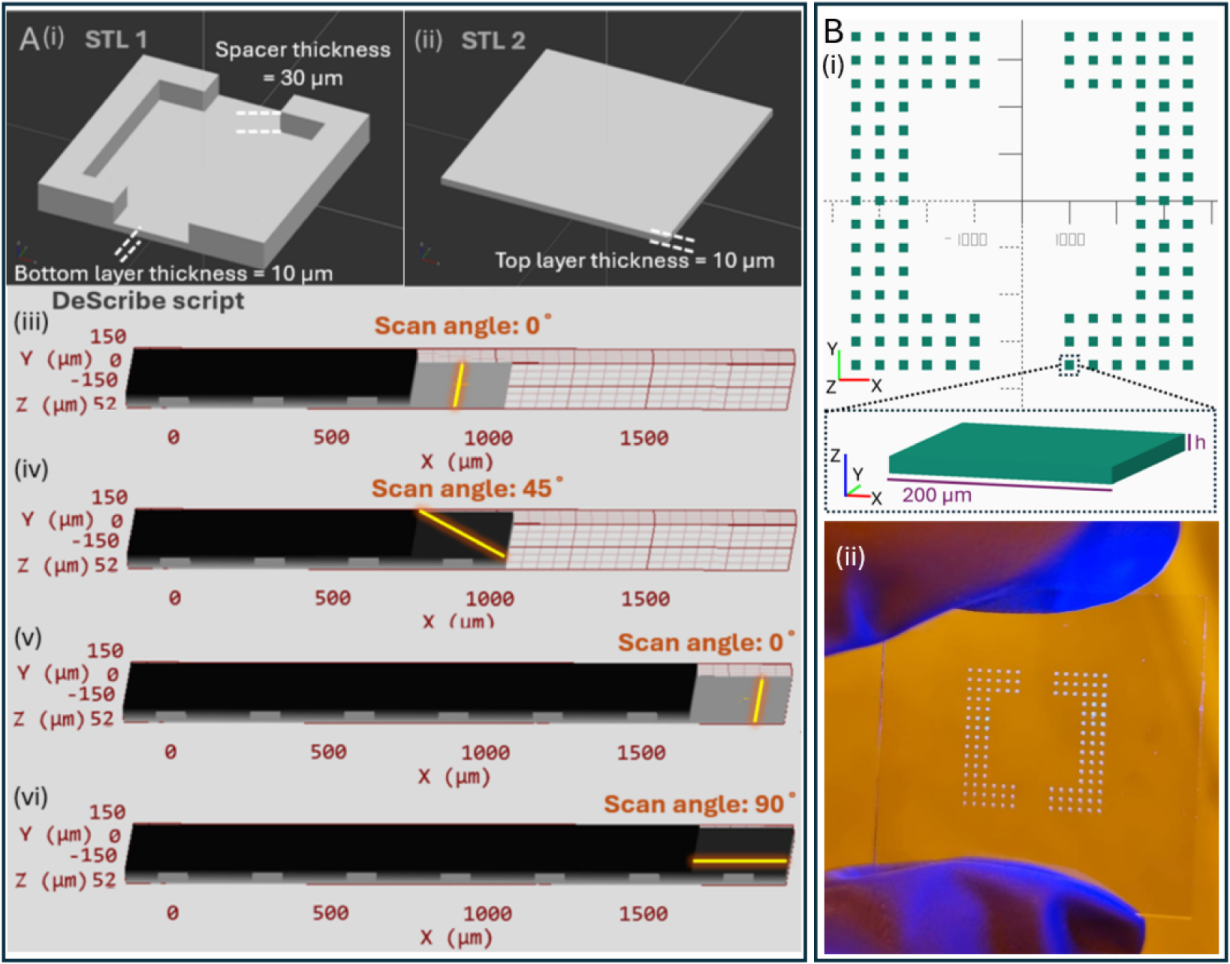
(A) Fabrication of TN cells with varying twist angles: (i) STL model defining the bottom alignment layer and spacers, (ii) STL model defining the top alignment layer, (iii−vi) exemplary *DeScribe* script printing steps: (iii) printing of a bottom alignment layer with scan angle 0°, (iv) printing of a top alignment layer with scan angle 45°, (v) printing of another bottom alignment layer with scan angle 0°, (vi) printing of another top alignment layer with scan angle 45°. (B) Fabrication of spacers used for separation of two substrates in cells combining 3D-nanoprinted and commercial alignment layers: (i) CAD model of the spacer array, with a central region of 5 × 5 mm, inset shows a unit spacer with lateral dimensions of 200 × 200 μm and arbitrarily defined height *h*, (ii) optical image of a 3D-nanoprinted spacer on a glass substrate.

## Abbreviations

LC: liquid crystal
2PP: two-photon polymerization
DLW: direct laser writing
TN: twisted nematic
POM: polarized optical microscopy
SEM: scanning electron microscopy
PGMEA: propylene glycol monomethyl ether acetate
IPA: isopropanol
